# Do the methods for cleaning the base of brackets used in indirect bonding interfere with adhesion to tooth enamel?

**DOI:** 10.1590/2177-6709.29.4.e242462.oar

**Published:** 2024-09-02

**Authors:** Carlos Eduardo de Paiva Campos Nogueira SIMÃO, Ana Luiza Ferreira da SILVA, Marcela Emílio de ARAÚJO, Sergei Godeiro Fernandes Rabelo CALDAS

**Affiliations:** 1 Federal University of Rio Grande do Norte, School of Dentistry, Department of Dentistry (Natal/RN, Brazil).; 2 Brazilian Dental Association (Natal/RN, Brazil).

**Keywords:** Shear strength, Orthodontic brackets, Orthodontics, corrective, Resistência ao cisalhamento, Braquetes ortodônticos, Ortodontia corretiva

## Abstract

**Objective:**

The objective of this study was to evaluate the shear bond strength of metal brackets bonded with indirect bonding, under different surface treatment protocols.

**Material and Methods:**

40 bovine teeth were randomly divided into four groups (n = 10), according to the type of surface treatment: G1 = 70% alcohol, G2 = air/water spray, G3 = 100-µm aluminum oxide blasting, G4 = direct boning. After drying, the standard Edgewise central incisor brackets were bonded with light-cured resin. The brackets were moved from the plaster models by means of a transfer tray made with condensation silicone, and bonded to the surface of the enamel with self-curing adhesive. The samples were submitted to shear tests by a universal test machine. Data were analyzed with SPSS 20.0 by the one-way ANOVA test and the Tukey post-test.

**Results:**

No statistically significant difference (*p*=0.174) was observed between the mean forces measured between the group for shear strength values of the groups during the test: G1 (5.33 MPa), G2 (3.52 MPa) and G3 (4.58 MPa).

**Conclusion:**

The bracket surface treatment protocols presented similarities in shear bond strength test. However, alcohol 70% and oxide blasting presented higher absolute values of resistance than the water group.

## RESUMO

**Objetivo:**

O objetivo desse trabalho foi comparar a resistência ao cisalhamento de braquetes metálicos colados após diferentes protocolos de tratamento de superfície durante a colagem indireta.

**Material e Métodos:**

40 dentes bovinos foram divididos aleatoriamente em quatro grupos (n=10), de acordo com o tratamento de superfície a que seriam submetidos: G1=álcool a 70%, G2=jatos de ar/água, G3=jateamento com partículas de óxido de alumínio de 100 µm, G4= colagem direta. Após a secagem, foi realizada a colagem dos braquetes de incisivo central Edgewise *standard* com resina fotopolimerizável. Os braquetes foram transferidos dos modelos de gesso por meio de uma guia confeccionada com silicone de condensação e colados à superfície do esmalte com o adesivo autopolimerizável. Após essa fase, foram submetidos ao teste de cisalhamento. Os dados foram analisados no *software* SPSS 20.0 pelo teste ANOVA de uma via e o pós-teste de Tukey.

**Resultados:**

Não foram encontradas diferenças estatisticamente significativas (*p*=0,174) entre as médias dos valores de resistência ao cisalhamento dos grupos durante o ensaio: G1 (5,33 MPa), G2 (3,52 MPa) e G3 (4,58 MPa).

**Conclusão:**

Os resultados dos testes com os protocolos de tratamento de superfície dos braquetes apresentaram similaridade. No entanto, os grupos com uso de álcool a 70% e de jateamento com partículas óxido de alumínio apresentaram valores absolutos de resistência maiores do que o grupo com jatos de água.

## INTRODUCTION

The development of the acid-etch technique^1^ allowed the use of composite resin in many fields of Dentistry. In Orthodontics, bracket bonding can be done by two techniques: direct and indirect. The first has only a clinical stage, in which the professional places the bracket directly on tooth surface. The second consists of one laboratory stage in which the brackets are located on the working model, and a clinical step in which the brackets are placed in the right position on tooth surface, with the help of a tray. The trays are made of fast-setting rubber materials, such as condensation silicone and hot glue.^2^ ^,^ ^3^

The indirect technique is used by many orthodontists because of its advantages, such as: reduction of clinical time, greater comfort for the patient, decreased stress for the operator and increased accuracy in bracket placement. It provides a view of the teeth on the model in all planes of space, allowing, mainly, a better positioning of the brackets.^4^ The indirect procedure is technically sensitive and can lead to reduced adhesion forces if the execution protocol is not carefully controlled.^5^ It also presents disadvantages, such as laboratory work time,^6^ higher costs, a greater number of steps and the interface between bonding resin and the adhesive applied to the tooth that can compromise adhesion. However, studies have reported that the adhesion strength obtained with indirect bonding may be sufficient for its clinical use.^2^

Indirect bonding creates a sensible interface when compared to direct bonding: the polymerized resin/adhesive interface, which can be a weak link,^7^ due to debris and remnants of the laboratory stage, impregnated in the polymerized resin from the base of the brackets, or even residues of insulating material and plaster and several contaminants.^8^ Few laboratory investigations study this interface, considering that the cleaning of these surfaces seems to be a decisive factor in the bond strength obtained in the indirect bonding protocol.

There is no defined protocol for cleaning this polymerized resin surface on the bracket base after bonding to the working model. Literature recommends using aluminium oxide jets.^5^ ^,^ ^9^ However, other authors claim that washing only with an air/water jet is enough to eliminate any trace of impurity.^7^ ^,^ ^10^ There are also reports in the literature that use only 70% alcohol to clean the bracket bases.^11^ ^,^ ^12^

However, no studies in the literature compared the various surface cleaning methods, aiming an adequate adhesion force for bonding orthodontic brackets. Therefore, the present study aimed to evaluate the shear bond strength of the metal brackets bonded using the indirect bonding technique with different surface treatment protocols, when compared to direct bonding. 

The hypothesis tested was that adhesive strength of metallic brackets bonded by indirect technique is influenced by the surface treatment of the bracket.

## MATERIAL AND METHODS

### SAMPLE PREPARATION

**Figure 1:**
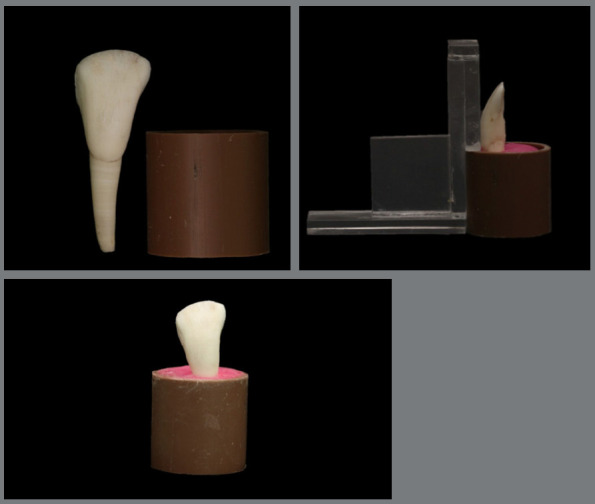
Sample preparation.

Forty bovine lower incisors with intact crowns were selected. The soft tissue adhered to the roots was removed with the aid of periodontal curettes. Then, the crowns were stored in distilled water at 4ºC until the specimens were prepared (ISO 11405)^13^, for a maximum period of one month. The crowns were then fixed in segments of polyvinyl chloride tubes 1 inch and 3 cm high with acrylic resin, up to the cementoenamel junction, standardizing the specimens. An acrylic positioning guide was made to align the vestibular surface of the teeth perpendicular to the tube’s base (Fig 1).

**Figure 2: f2:**
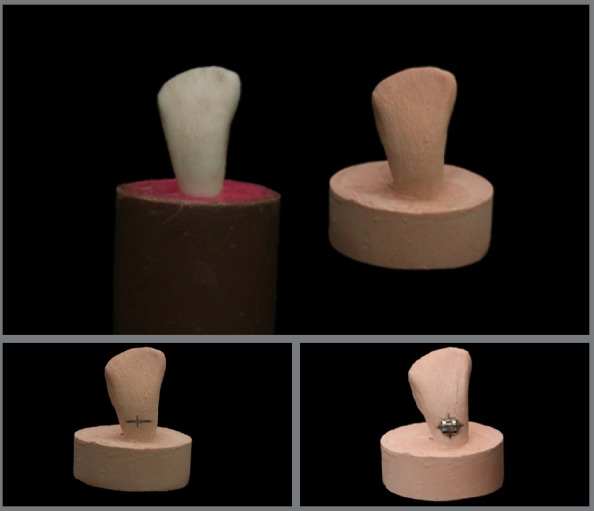
Positioning the metal bracket on the plaster model.

The metallic brackets (Morelli, Sorocaba, São Paulo, Brazil) used in all groups were lower incisor standard Edgewise. The brackets of indirect technique groups were positioned on the working model using a thin layer of Transbond™ XT Light Cure Adhesive (3M Unitek, Sumaré, São Paulo, Brazil) (Fig 2). The brackets were positioned in the flattest region on the crown’s buccal surface, 5 mm above the edge of the specimen, with a manual pressure of 500 gF, standardized with the aid of a tensiometer (Morelli, Sorocaba, São Paulo, Brazil). Excessive bonding material was removed with explorer instrument no. 5.

[Fig f2]

After removing the excesses, each bracket was photoactivated for 6 seconds (3 seconds on the mesial surface and 3 seconds on the distal surface) with a high-power LED light curing device with an irradiance of 3200 mW/cm^2^ (VALO Ortho, Ultradent, South Jordan, Utah, USA). This device was calibrated at the beginning of the experiment with a radiometer, ensuring a standard light intensity. 

**Figure 3:**
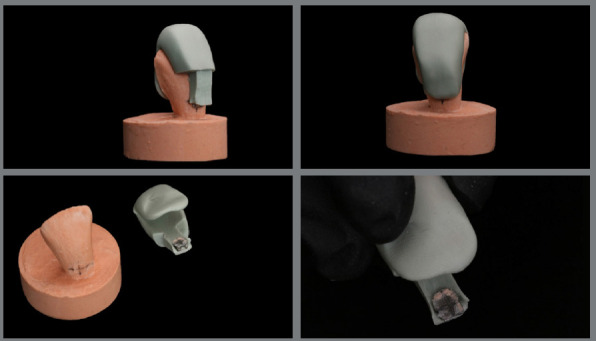
Confection of the individual transfer tray with condensation silicone.

Immediately after bonding the brackets, transfer trays were made with condensation silicone (Zetaplus, Zhermack, Badia Polesine, Rovigo, Italy). The trays were individualized and made in a certain way to cover the incisal and buccal surfaces of all teeth up to their middle third, leaving their cervical portion exposed. After the material had been set, the plaster models and the respective silicone trays were then immersed in a container with water at room temperature for 12 hours, to facilitate the removal of the transfer tray/bracket (Fig 3). 

**Figure 4:**
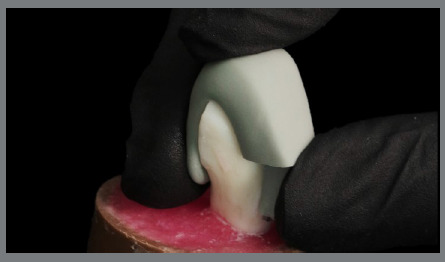
Tray/bracket set.

**Figure 5:**
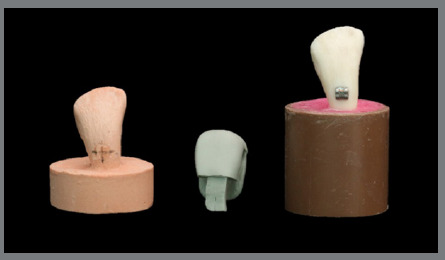
Sample made.

To allow greater flexibility and easy removal of the trays during the clinical bonding of brackets, reliefs were made in the silicone with the aid of a scalpel and a no. 15 blade. The thirty specimens and their respective plaster models were randomly divided into three groups (n = 10) according to the surface treatment (Figs 4 and 5).

In G1, a microbrush soaked with 70% liquid alcohol was rubbed against the base of brackets and a 2 minutes period was waited to allow the remaining alcohol to evaporate from the bases and dry completely. G2 was subjected to a water/air spray from the triple syringe, with the jet applied for 3 seconds on each bracket base. Then, 5 second air jets were used on each of the bases to dry them. In G3, sandblasting was performed with 100-µm microparticles aluminium oxide, with the jet directed perpendicular for 3 seconds over each bracket base. Subsequently, the bases were rinsed with water and dried with air jets from the triple syringe, 5 seconds for each bracket. 

Immediately before transferring the brackets to the teeth crowns, the enamel preparation was carried out with prophylaxis using a rubber cup and putty (pumice + water) with the aid of a low speed handpiece, for 10 seconds on each tooth. Each rubber cup was used for a maximum of ten crowns. The crowns were rinsed with running water, to remove the paste, and dried with air jets for 10 seconds. 

Acid etching of the tooth surfaces was sequentially performed with Ultra-Etch™ phosphoric acid (Ultradent, South Jordan, Utah, USA) at 35% for 15 seconds, rinsed with a water jet for 10 seconds and dried with air jet for another 10 seconds, according to manufacturer’s instructions. 

Transferring the brackets from the tray to teeth crowns was performed using a chemically activated resin (Maximum Cure, Reliance, Itasca, Illinois, USA). This material consists of two bottles, one of which is Maximum Cure Sealant part A with fluoride, and the other is Maximum Cure Sealant part B. According to manufacturer’s recommendations, the bond is applied directly to the tooth surface by an operator using a microbrush. Simultaneously, another operator applies a thin layer of adhesive to the bracket base. 

After applying the chemically activated resin to the tooth surface and trays, the tray was immediately positioned on the tooth, applying a manual pressure for 60 seconds by the operator. After that time, the specimen was left with the transfer tray in position for 5 minutes before proceeding with removal. The removal was performed with the aid of a fine-tipped instrument, leaving the brackets bonded to the teeth. 

The G4 (control) was bonded with direct technique. It followed the same preparation: enamel prophylaxis with a rubber cup and putty (pumice + water), followed by the same conditioning protocol used before. Then, the brackets were directly positioned on the tooth surface, in the same position of other groups, bonded with Transbond™ XT Light Cure Adhesive and photoactivated for 6 seconds using the same protocol. 

### AGING

The specimens were distributed to simulate aging in four plastic containers, according to the group they belonged, in distilled water for 4 months at 37ºC.

### SHEAR BOND STRENGTH

**Figure 6:**
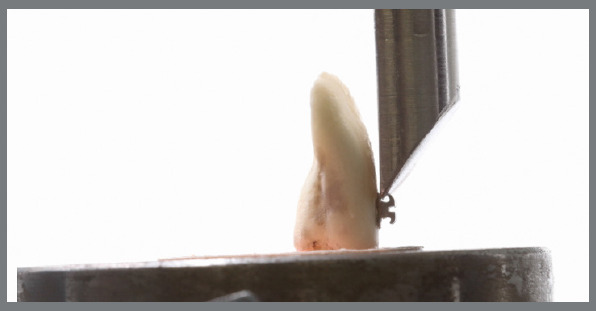
Mechanical shear bond strength test.

After this period, samples were submitted to mechanical tests to evaluate the adhesion strength in each group. Tests were carried out by shear test using an Oswaldo Filizola universal mechanical testing machine (Oswaldo Filizola, São Paulo, Brazil), model AME5k, in which a metallic device was adapted to fixate the specimen, in order to keep bracket/enamel interface perpendicular to the horizontal plane (parallel to the shear force). Another chisel-shaped device was attached to the machine’s load cell (500N) and applied in the occlusal-gingival direction to the bracket/enamel interface, at a constant speed of 1 mm/min until fracture occurred (Fig 6).

### ADHESIVE REMNANT INDEX (ARI)

To complement this study, all teeth were assessed after bracket detachment. Two calibrated operators, with the aid of a stereomicroscope (Nikon SMZZ800, Tokyo, Japan) and 25x magnification, evaluated the types of bond failure and classified them according to the Adhesive Remnant Index (ARI), proposed by Årtun and Bergland^14^: score 0 (no adhesive left on enamel), score 1 (less than half of the adhesive left on the enamel), score 2 (more than half of the adhesive left on enamel) and score 3 (all the resin left on enamel).

### DATA ANALYSIS

Data obtained in the shear bond strength test were organized, tabulated, and analyzed using the Jamovi^®^ software (Jamovi Stats Open Now, Sydney, Australia). Results were submitted to the analysis of Shapiro-Wilk test for normality. Shear bond strength results were evaluated descriptively and compared through ANOVA one-way test and Tukey’s test. The ARI results were analyzed descriptively. In all tests used, the significance level adopted was 95%.

## RESULTS

Shapiro-Wilk test was performed for each group and indicated that data were normally distributed (*p*>0.05). The ANOVA one-way results for the experimental conditions are presented in Table 1. A significant difference in the comparison between the experimental groups for the studied factor was shown by the test (*p*=0.002). 

**Table 1:** Results of one factor ANOVA for the “surface treatment” factor, according to bond strength (p < 0.05).

Variable	df1	df2	F	p-value
Surface treatment	3	18.7	6.97	0.002*

*Statistically significant difference at the 5% level.

The adhesion force values (MPa) obtained in the shear test by the four groups in this research are described in Table 2. Tukey’s test (5%) for the comparison between groups demonstrated that indirect bonding protocols resulted in no statistically significant differences between the groups, just when compared to direct bonding group.

**Table 2:** Bond strength values (MPa) according to the experimental groups (n=10).

Group	Surface treatment	Mean	SD
G1 - IB	70% alcohol	5.33^A^	2.41
G2 - IB	Water/Air jet	3.52^A^	2.49
G3 - IB	Sandblasting aluminum oxide 100μm	4.89^A^	1.49
G4 - DB	-	16.94^B^	6.03

IB: Indirect bonding; DB: Direct bonding; SD: Standard deviation; Tukey test (p <0.05%). Equal superscripts letters indicate statistical similarity.

The descriptive results of bracket bases assessment about ARI are shown in Table 3. Most specimens from G3 had scores of 2, while G1 and G2 had the same number of specimens with scores of 0 and 2. The direct bonding group had most of the samples on score 0.

**Table 3:** Distribution of Adhesive Remnant Index (ARI) scores.

Groups	ARI values				Mean
	0	1	2	3	
G1	3	2	3	2	1.4
G2	3	3	3	1	1.2
G3	1	2	4	3	1.9
G4	6	1	1	2	0.9

G1 - 70% alcohol; G2 - Water/air; G3 - Aluminum oxide sandblasting; G4 - Direct bonding.

## DISCUSSION

The results showed in the present study rejected the hypothesis that the surface treatment influences the adhesive strength of metallic brackets bonded by indirect technique to enamel.

The data found in the literature that served as a comparison parameter for this study were the values proposed by Reynolds^15^ in his 1975 review, which presented a force range of 5.9 to 7.8 MPa as necessary for brackets to be accepted as clinically successful results for orthodontic purposes. In a recent study, evaluating the relevant literature on the subject, the average values ​​of bracket adhesion to enamel were around 14.05 MPa with a standard deviation of 6.52 MPa (range 7.53 to 20.57 MPa).^16^ The average values obtained by indirect bonding were 5.33 MPa in Group 1 (70% alcohol), 3.52 MPa in Group 2 (water/air jet), and 4.89 MPa in Group 3 (aluminium oxide sandblasting). These values are below the ideal values proposed by the studies cited.^15^ ^,^ ^16^ Statistical difference was only observed when comparing Groups 1, 2 and 3 with Group 4 (direct bonding - control group) which had an average value of 16.94 MPa, more than double that recommended by Reynolds.^15^

Researchers advocate cleaning the surface of brackets with an air/water jet as an indirect bonding protocol.^7^ ^,^ ^10^ ^,^ ^17^ However, in this study, Group 2 (air/water jet) had the lowest absolute values, suggesting that this technique is unable to remove impurities present at the bracket base during transfer, in contrast to the findings in the literature.

Regarding to sandblasting with aluminium oxide at the brackets base, the literature reports that it promotes an increase in the adhesion strength of composites to metallic brackets.^18^ ^,^ ^19^ A study showed that sandblasting with aluminium oxide improved all adhesiveness values at the resin/bracket interface.^20^ However, the blasting protocol used in this research does not seem to provide additional benefits, since it did not show significantly higher adherence values, when compared to other groups. 

It is important to highlight that many variables are involved in the application of the aluminium oxide jet, such as: different abrasive particle size,^21^ tip diameter, abrasive air pressure,^22^ angle of application, and blasting time. Therefore, when comparing the results of the different investigations, it must be considered that different blasting protocols can be used, as well as different bonding materials and mechanical tests that can lead to different results.^23^ It is suggested that the blasting protocol promoted similar cleaning of the bracket base, allowing bonding of the adhesive system with the bracket base resin.

Although sandblasting with aluminium oxide is the most recommended surface treatment found in literature, the study group that used 70% alcohol was the one that obtained the highest average value of shear strength. This demonstrates that alcohol can be a strong substitute for oxide blasting, given its performance comparable to Group 3, although still below that recommended by Reynolds.^15^ Thus, 70% alcohol appears to be an excellent option for orthodontists to adopt in their clinical protocols for indirect bonding, due to its lower cost and easy availability.

If the minimum and maximum values of the adhesion forces found in this test are analyzed, it is possible to notice a variability of the results in all groups. This fact can be explained by the use of chemical curing adhesive in bonding, as the polymerization begins as soon as the two components of the material gets in contact. Therefore, it is inevitable that the material in the bracket bases that were loaded first will be in a more advanced state of polymerization than the material in the brackets that were loaded at the end of the procedure. This uneven rate of polymerization can increase air inclusions, which can cause a considerable reduction in adhesion strength.^2^

Another relevant factor in this study was the type of failure that occurred when removing the bracket from the enamel surface during the shear test. Bonding failure can occur in three ways: at the bracket/resin interface, at the enamel/resin interface, or both - each with its advantages and disadvantages.^24^ Bracket failure at the bracket/resin interface (high ARI scores) is advantageous as it leaves the enamel surface relatively intact and indicates a reduced risk of enamel damage during debonding procedure, which may be beneficial for the patient.^25^ ^,^ ^26^

However, considerable chair time is required to remove residual adhesive, with the increased possibility of damaging the enamel surface during the cleaning process. On the other hand, when brackets fail at the enamel/resin interface (low ARI scores), less residual adhesive remains, but the enamel surface can be damaged when the failure occurs in this way.^20^ ^,^ ^27^ ^,^ ^28^

Results of ARI scores from this study indicated that brackets bonded with either surface treatment system showed a similar range of bond failures (Table 3). Although the four groups had specimens in all types of failure, the mean ARI of all groups was closer to the lowest scores (ARI<2). These results show that there was a tendency for failures to occur more at the enamel/resin interface, with less resin remaining adhered to the enamel after detaching the brackets. 

During the analysis of descriptive ARI results, it was possible to observe that the average was close to scoring 2 and that only 8.3% of the specimens from Group 3 presented scores close to 0. This allows inferring that, when the brackets are submitted to sandblasting, there is a better chemical union at the enamel/resin interface, therefore, during the debonding process, a greater amount of resin remains adhered to the tooth surface than to the base of the bracket.

Thus, the laboratory study was chosen as a base for suggesting better protocols for clinicians. Despite the limitations inherent to *in vitro* studies in presenting the same types of protocol that would make it possible to compare our results, it is necessary to remember that, during indirect bonding, there is the formation of an interface between the polymerized resin at the brackets base and the adhesive applied to the tooth surface at the time of bonding procedure, which may compromise the adhesion strength if the surface if not cleaned.

## CONCLUSION

Based on the results obtained and the methodology used, it is possible to conclude that:

» There were no statistically significant differences between the means of shear bond strength values presented by the different treatment surfaces. However, the low means suggests a higher chance of bond failure.
» The direct bonding had better means of shear bond strength, when compared to indirect bonding.
» ARI scores were very similar for all groups, with the highest average obtained by G3.
